# Radiomic models based on magnetic resonance imaging predict the spatial distribution of CD8^+^ tumor-infiltrating lymphocytes in breast cancer

**DOI:** 10.3389/fimmu.2022.1080048

**Published:** 2022-12-19

**Authors:** Seung Hyuck Jeon, So-Woon Kim, Kiyong Na, Mirinae Seo, Yu-Mee Sohn, Yu Jin Lim

**Affiliations:** ^1^ Graduate School of Medical Science and Engineering, Korea Advanced Institute of Science and Technology, Daejeon, Republic of Korea; ^2^ Department of Pathology, Kyung Hee University College of Medicine, Kyung Hee University Medical Center, Seoul, Republic of Korea; ^3^ Department of Radiology, Kyung Hee University College of Medicine, Kyung Hee University Medical Center, Seoul, Republic of Korea; ^4^ Department of Radiation Oncology, Kyung Hee University College of Medicine, Kyung Hee University Medical Center, Seoul, Republic of Korea

**Keywords:** immunophenotype, CD8+ T cells, radiomics, breast cancer, magnetic resonanace imaging

## Abstract

Infiltration of CD8^+^ T cells and their spatial contexture, represented by immunophenotype, predict the prognosis and therapeutic response in breast cancer. However, a non-surgical method using radiomics to evaluate breast cancer immunophenotype has not been explored. Here, we assessed the CD8^+^ T cell-based immunophenotype in patients with breast cancer undergoing upfront surgery (n = 182). We extracted radiomic features from the four phases of dynamic contrast-enhanced magnetic resonance imaging, and randomly divided the patients into training (n = 137) and validation (n = 45) cohorts. For predicting the immunophenotypes, radiomic models (RMs) that combined the four phases demonstrated superior performance to those derived from a single phase. For discriminating the inflamed tumor from the non-inflamed tumor, the feature-based combination model from the whole tumor (RM-whole_FC_) showed high performance in both training (area under the receiver operating characteristic curve [AUC] = 0.973) and validation cohorts (AUC = 0.985). Similarly, the feature-based combination model from the peripheral tumor (RM-peri_FC_) discriminated between immune-desert and excluded tumors with high performance in both training (AUC = 0.993) and validation cohorts (AUC = 0.984). Both RM-whole_FC_ and RM-peri_FC_ demonstrated good to excellent performance for every molecular subtype. Furthermore, in patients who underwent neoadjuvant chemotherapy (n = 64), pre-treatment images showed that tumors exhibiting complete response to neoadjuvant chemotherapy had significantly higher scores from RM-whole_FC_ and lower scores from RM-peri_FC_. Our RMs predicted the immunophenotype of breast cancer based on the spatial distribution of CD8^+^ T cells with high accuracy. This approach can be used to stratify patients non-invasively based on the status of the tumor-immune microenvironment.

## Introduction

According to recent cancer statistics, breast cancer is the most common type of cancer in women (1). Breast cancer is a heterogenous entity with diverse biological characteristics associated with prognosis and therapeutic response (2). Although breast cancer has been known to exhibit less immunogenic potential, the prognostic and predictive significance of tumor-infiltrating lymphocytes (TILs) has been suggested (3); a higher TIL infiltration was associated with better response to neoadjuvant chemotherapy (NACT) and longer patient survival (4). The level of TILs was recently reported to be a strong predictor of response to immunotherapy in the basal-like subtype of breast cancer (5, 6).

Among TILs, CD8^+^ T cells have been known to play a crucial role in the tumor-immune microenvironment. Breast cancer with high number of TILs contained elevated number of tissue-resident memory CD8^+^ T cells, which were associated with improved survival and thought to play local anti-tumor activity (7, 8). Also, the infiltration of CD8^+^ T cells is reportedly correlated with longer survival (9–11). Gruosso et al. showed that the spatial distribution of CD8^+^ T cells, evaluated in both tumor core and invasive margins, was related to distinct biological and prognostic features (12). The spatial contexture of CD8^+^ T cells within the breast cancer tissues was also correlated with differential response to anti-PD-1 treatment, suggesting therapeutic implications of the immunophenotype (13). However, in real-world clinics, the spatial distribution of CD8^+^ T cells cannot be assessed until tumors are surgically resected.

Radiomics utilizes numerous quantitative features extracted from medical images to determine clinical and prognostic outcomes. Based on the image data, radiomic models (RMs) that predicted survival, lymph node metastasis, or molecular subtypes of breast cancer have been developed (14–16). Since dynamic contrast-enhanced magnetic resonance imaging (DCE-MRI) is usually acquired for the initial diagnosis of breast cancer, RMs using the DCE-MRI can be clinically used. Although a few studies have predicted the level of TILs using RMs (17–19), radiomics has not been applied to discriminate the spatial contexture of the tumor-immune microenvironment.

Here, we developed MRI-derived RMs to predict breast cancer immunophenotype based on the spatial distribution of CD8^+^ T cells. The combined data from each phase of DCE-MRI were used to improve the predictive performance. Our results may provide a non-invasive tool for stratifying patients based on the intratumoral immune response.

## Materials and methods

### Study population

This study analyzed two cohorts of patients with breast cancer: 1) upfront surgery and 2) NACT cohorts. The surgery cohort consisted of 182 patients who underwent curative surgery between January 2016 and February 2020 with the following criteria: (1) without distant metastasis at diagnosis; (2) no use of neoadjuvant therapy; (3) surgical specimens available for the evaluation of tumor immunophenotypes; (4) MRI scans at initial diagnosis available according to the institution’s protocol. Then, the surgery cohort was randomly divided into training and validation cohorts in a 3:1 ratio. The NACT cohort included 64 patients who underwent NACT followed by surgery between March 2013 and March 2022 with the following criteria: (1) no distant metastasis at diagnosis; (2) MRI scans available according to the institution’s protocol. The baseline characteristics of each cohort are described in **Supplementary Tables 1**, **2**. NACT was considered in selected cases: 1) inoperable disease status, such as bulky or matted ≥ cN2 and cT4 tumors; 2) multifocal or large primary tumor relative to breast size, which makes breast conservation difficult; and 3) HER2-positive or basal-like with ≥cT2 and/or cN+ status. The NACT regimens for HER2-negative cases included doxorubicin/cyclophosphamide (AC; n = 1), AC followed by weekly paclitaxel (n = 40), and AC followed by docetaxel every 3 weeks (n = 7). For HER2-positive cases, AC followed by paclitaxel/trastuzumab (n = 6) or docetaxel/carboplatin/trastuzumab/pertuzumab (n = 12) regimen was considered. The study protocol of this study was reviewed and approved by the Institutional Review Board of Kyung Hee University Hospital (2020-12-014).

### Pathologic preparation

We used formalin-fixed and paraffin-embedded tissue samples of breast cancer pathologically confirmed after surgical resection. Hematoxylin and eosin (H&E)-stained slides were examined by two pathologists with expertise in breast cancer (S-WK and KN) to confirm the histological tumor diagnosis and select the appropriate representative slides for subsequent analyses. Only samples with greater than 10% tumor cellularity were used for further analysis. Immunohistochemical (IHC) staining was performed using 4-μm-thick sections prepared from whole-section blocks and carried out on a Bond-III immunostainer (Leica Biosystems, Newcastle, UK) according to the manufacturer’s instructions. Tissue sections were deparaffinized, antigen-retrieved, and then incubated for 15 min at ambient temperature with a monoclonal antibody against CD8 (1:400, M7103, Dako, Carpinteria, CA). The nuclei were counterstained with hematoxylin. Tonsil tissue was used as external positive control.

### Immunophenotype assessment and quantification of lymphocytes

**Figure 1:**
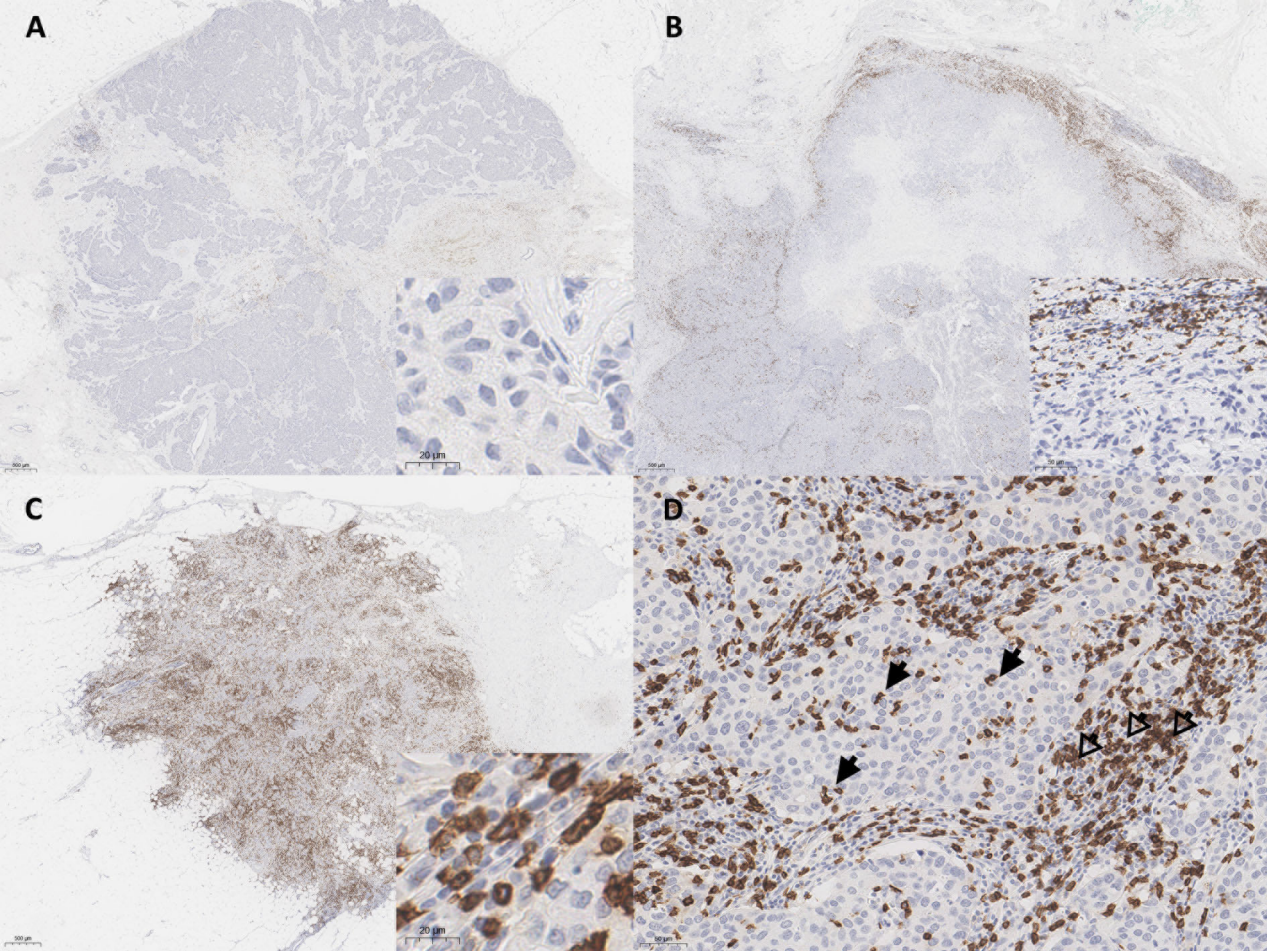
Representative whole slide images of CD8^+^ T cell spatial immunophenotypes in breast cancer; **(A)** immune-desert, **(B)** immune-excluded, and **(C)** immune-inflamed. **(D)** In high power view, intratumoral tumor-infiltrating lymphocytes (TILs) (arrows) and stromal TILs (open arrows) are noted. The blue color is the hematoxylin counterstain. Original magnification, A-C, scanning view D, ×200; inset, A, C, ×400, B, ×200.

The density and spatial distribution of CD8^+^ TILs were evaluated using IHC-stained whole-sectioned slides. The tumor center (TC) and invasion front (IF) of the breast cancer lesions were separately evaluated. The IF was defined as the most progressed cancer cells on the advanced edge of the tumor. The hotspots in the TC and IF were selected at low magnification, individually marked on H&E slides, and transferred on IHC-stained whole-sectioned slides. Then, the CD8-stained slides were scanned using an AperioScanScope (Aperio Technologies, Vista, CA). Scanned whole-slide images were analyzed using the open access image analysis software QuPath as previously described (20). Briefly, the *Simple tissue detection* tool was used to create an annotation of the tissue region to be analyzed, and the tumor border of each sample was manually outlined using the *Polygon* tool, creating three different regions: outer margin and inner margin (referred to as IF), and TC. Next, the *Cell detection* tool using a built-in cell segmentation algorithm and the *Add smoothed features* (25 µm) tool calculated a new measurement by taking a weighted average of cell measurements within the 25-µm range, whereby the image was segmented homogeneously. The *Intensity feature* in the *Detection classifier* was used to distinguish between positively and negatively stained cells. The density of CD8^+^ TILs was automatically enumerated in three hotspots from each TC and IF, defined as the number of positively stained lymphocytes/mm^2^. Based on the distribution pattern of the CD8^+^ TILs, tumor immunophenotype was defined as mentioned previously (21), which was classified into immune-desert (no immune cell infiltration in invasive tumor margin), immune-excluded (immune cells aggregating only in invasive tumor margin) and inflamed (prominent immune infiltrates in the tumor core through invasive tumor margin) subtypes (**Figures 1A–C**).

Stromal TILs were also separately counted from the hotspots in each case. The stromal TIL was defined as a dispersed lymphocyte in the stroma between the cancer cells, but without direct contact with cancer cells (**Figure 1D**) (21). The QuPath results for all samples were confirmed independently by two pathologists (S-WK and KN), who ensured the software’s accuracy, and the QuPath results were then used for quantitative analysis.

### MRI and region of interest (ROI)

All MRI scans were acquired with a 3-T scanner (Achieva; Philips Healthcare, Best, the Netherlands) using a breast coil (SENSE-Breast 7TX, Philips Healthcare, Best, the Netherlands) in the prone position. Axial 3-dimensional DCE-MRI was obtained using the following parameters: FOV = 496 x 316 mm, TR/TE = 4.3/2.25 msec, flip angle = 12°, spatial resolution = 0.6 x 0.6 x 2 mm, section thickness = 4 mm. Gadobenate dimegumine (0.1 mmol/kg; Multihance, Bracco Imaging, Milan, Italy) was injected, and pre-contrast and four consecutive post-contrast images with 74-second intervals were acquired (hereafter referred to as DCE_1_, DCE_2_, DCE_3_, and DCE_4_). The tumor ROI was delineated on the subtraction images, obtained by subtracting the pre-contrast images from the second post-contrast images on a pixel-by-pixel basis, by a radiologist specializing in breast imaging for 10 years (MS). The tumor periphery was defined as the inner 2-mm rim of the whole tumor.

### Image preprocessing and radiomic feature extraction

After image preprocessing, 64 texture features calculated from the first-order statistics, gray level co-occurrence matrix (GLCM), gray level run length matrix (GLRLM), gray level size zone matrix (GLSZM), and neighbor gray tone difference matrix (NGTDM) were obtained from the whole tumor or peripheral ROI. The mathematical definitions of the 64 texture features are detailed in **Supplementary Material**. In addition, the preprocessed images were wavelet-transformed with the following wavelet filters to generate 12 transformed images: Daubechies 2 (ratio = 1/2, 2/3, 3/2, or 2), Coiflets 1 (ratio = 1/2, 2/3, 3/2, or 2), and Symlets 4 (ratio = 1/2, 2/3, 3/2, or 2). The 64 texture features were extracted from each wavelet-transformed image.

### Filtering of radiomic features

Each radiomic feature group (RFG) comprised 833 radiomic features (tumor volume, 64 texture features, and 768 wavelet features) extracted from either whole tumor or peripheral ROI on a single MRI sequence. The RFGs extracted from DCE_1_, DCE_2_, DCE_3_, and DCE_4_ were termed RFG_1_, RFG_2_, RFG_3_, and RFG_4_, respectively. In addition to the aforementioned original ROIs, a radiation oncologist (SHJ) segmented tumors from 20 randomly selected patients to select stable radiomic features. Informative radiomic features with an intraclass correlation coefficient (ICC) lower than 0.8 were filtered out from each RFG, as previously described (22). Next, the area under the receiver operating characteristic curve (AUC) value for each radiomic feature was calculated to predict tumor immunophenotype; the top 50 radiomic features with the highest AUC values were selected as informative features. For RFGs with less than 50 stable radiomic features, all stable radiomic features were used to develop RMs.

### Development and validation of RMs

**Figure 2:**
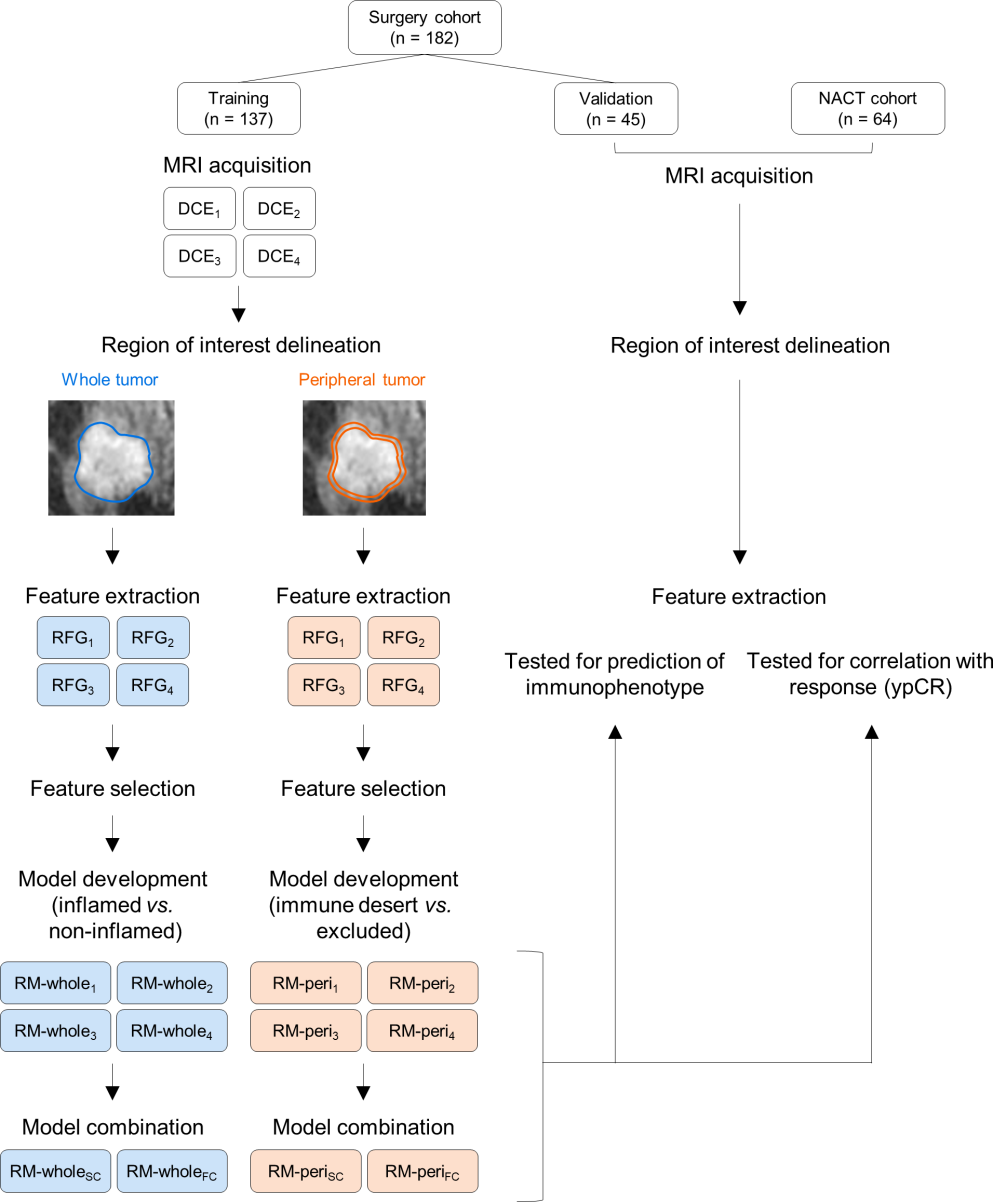
The study workflow for developing radiomic models and their validation. NACT, neoadjuvant chemotherapy; MRI, magnetic resonance imaging; DCE, dynamic contrast enhanced; RFG, radiomic feature group; RM, radiomic model.

Two types of RMs were developed in this study; the first one was to discriminate inflamed tumors from immune-desert or excluded tumors using RFGs from the whole tumor (RM-whole), and the second one was to discriminate immune-desert tumors from immune-excluded tumors using RFGs from the tumor periphery (RM-peri). For both modeling types, least absolute shrinkage and selection operator (LASSO) regression was used to select the radiomic features and build score-based models from the training cohort with 5-fold cross-validation. The tuning parameter (λ) with the minimum cross-validation error was selected. The regression was performed using the R package *glmnet* with the following parameters: alpha = 1 and maxit = 10^4^. The radiomic score was defined by the linear combination of the selected radiomic features and their respective coefficients. We combined the RMs to increase the prediction power using LASSO regression with the two methods. The score-combined model (SC) was developed using scores from the four individual RMs as input. On the other hand, the feature-combined model (FC) utilized the entire radiomic features selected in the four RMs for the combination. The parameters of the LASSO regression were the same as the primary modeling parameters. All RMs were developed from the training cohort and tested using the validation cohort. Additionally, the developed RMs were tested to evaluate the correlation with pathologic response in the NACT cohort. The overall flow of this study is represented in **Figure 2**.

### Clinical outcomes

Overall survival was defined as the time duration from surgery to death due to any cause. Disease-free survival was defined as the duration from surgery to clinical detection of recurrence. Pathologic complete response (ypCR) to NACT was defined as no residual tumor on the surgical specimens of the primary tumor and lymph nodes.

### Statistical analyses

The ICC values for inter-rater variability were calculated using a two-way random effects model. Kaplan-Meier curves were compared using the Cox proportional hazards model. AUC values of the models were calculated using the *pROC* R package and compared using Delong’s test. The association between two continuous variables was assessed using the Pearson’s correlation test. The continuous variables were compared using Student’s *t*-test or one-way analysis of variance (ANOVA) followed by *post-hoc* Tukey’s test, as appropriate. The optimal cutoff values were determined to maximize the sum of sensitivity and specificity of each model. For multiple comparisons, *P*-values were adjusted using Holm’s method. All statistical analyses were performed using R software version 4.1.0 (https://www.r-project.org).

## Results

### Association between immunophenotype and clinical parameters

In the upfront surgery cohort, 67 (36.8%), 30 (16.5%), and 85 (46.7%) had immune-desert, excluded, and inflamed phenotypes, respectively. As expected, inflamed tumors exhibited a remarkably higher stromal CD8^+^ cell density at the TC than other immunophenotypes (**Supplementary Figure 1A**), and immune-desert tumors showed a significantly lower stromal CD8^+^ cell density at the tumor periphery than others (**Supplementary Figure 1B**). The proportion of the inflamed phenotype was higher in HER-2 enriched and basal-like subtypes than in luminal subtypes (**Supplementary Figure 2**). The immune-excluded phenotype was associated with a higher rate of larger tumor size (*P* = 0.003), lymphatic invasion (*P* = 0.019), and higher histologic grade (*P* < 0.001) (**Supplementary Table 3**).

Next, we examined the relationship between stromal TIL density and CD8^+^ T cell-based immunophenotype. The stromal TIL density was positively associated with both central and peripheral stromal CD8^+^ cell densities (**Supplementary Figures 3A, B**). The stromal TIL density was lowest (0% except for one case) in cases with the immune-desert phenotype, while there was no significant difference in the stromal TIL density between immune-excluded and inflamed phenotypes (**Supplementary Figure 3C**). The immune-excluded feature was associated with worse progression-free and overall survival (*P* = 0.03 and 0.04, respectively) (**Supplementary Figures 4A, B**). However, the stromal TIL density was not significantly associated with survival outcomes (**Supplementary Figures 4C, D**). These data suggest that the spatial contexture of CD8^+^ TILs may provide additional clinical value to the stromal TIL density.

### Discrimination between inflamed and non-inflamed phenotypes using whole-tumor radiomics

**Figure 3:**
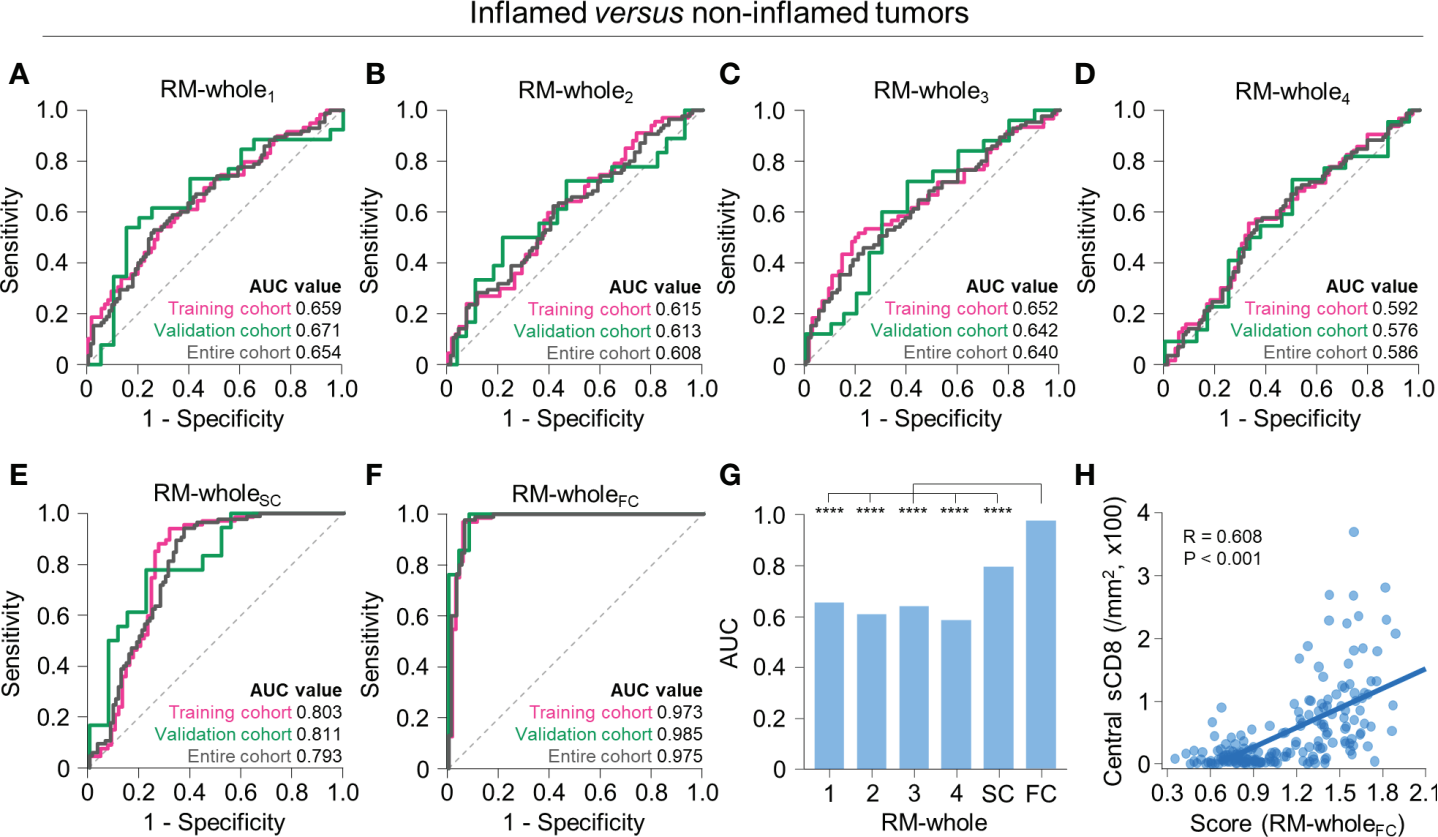
Radiomic models to predict immune-inflamed *versus* non-inflamed immunophenotypes on the basis of radiomic features from the whole tumor. **(A–F)** The receiver operating characteristic curves of RM-whole_1_ **(A)**, RM-whole_2_ **(B)**, RM-whole_3_ **(C)**, RM-whole_4_ **(D)**, RM-whole_SC_ **(E)**, and RM-whole_FC_ **(F)** for discriminating between immune-inflamed and non-inflamed tumors in the training (*pink*), validation (*green*), and entire cohorts (*gray*). **(G)** The comparison of the AUC values of the six radiomic models derived from the whole tumor. **(H)** The correlation between the radiomic score obtained from RM-whole_SC_ and the central stromal CD8^+^ T cell density. Statistical analyses were performed using Delong’s test **(G)** and Pearson’s correlation test **(H)**. ^****^ *P* < 0.0001. RM, radiomic model; AUC, area under the receiver operating characteristic curve; sCD8, stromal CD8^+^ T cells.

Using radiomic features from the whole tumor, we first developed RMs to predict whether the tumor has an inflamed or non-inflamed phenotype (immune-desert or excluded). Using a single RFG, the regression model selected 4–7 radiomic features (**Supplementary Table 4**). The performance of the model was highest for RM-whole_1_ (training, AUC = 0.659; validation, AUC = 0.671) and lowest for RM-whole_4_ (training, AUC = 0.592; validation, AUC = 0.576) (**Figures 3A–D**). For the RM developed using scores from the four RMs (RM-whole_SC_), the AUC values were 0.803 and 0.811 in the training and validation cohorts, respectively (**Figure 3E**). Of note, the RM developed using features selected from the four RMs (RM-whole_FC_) resulted in AUC values of 0.973 and 0.985 in the training and validation cohorts, respectively (**Figure 3F**). The selected features in the combined models are presented in **Supplementary Table 5**. The AUC value of RM-whole_FC_ in the entire upfront surgery cohort was significantly higher than that in any of the individual RMs and RM-whole_SC_ (*P* < 0.05 for all comparisons; **Figure 3G**). In addition, the central density of stromal CD8^+^ cells was positively correlated with the score from RM-whole_FC_ (*P* < 0.001; **Figure 3H**).

### Discrimination between immune-desert and excluded phenotypes using tumor periphery radiomics

**Figure 4:**
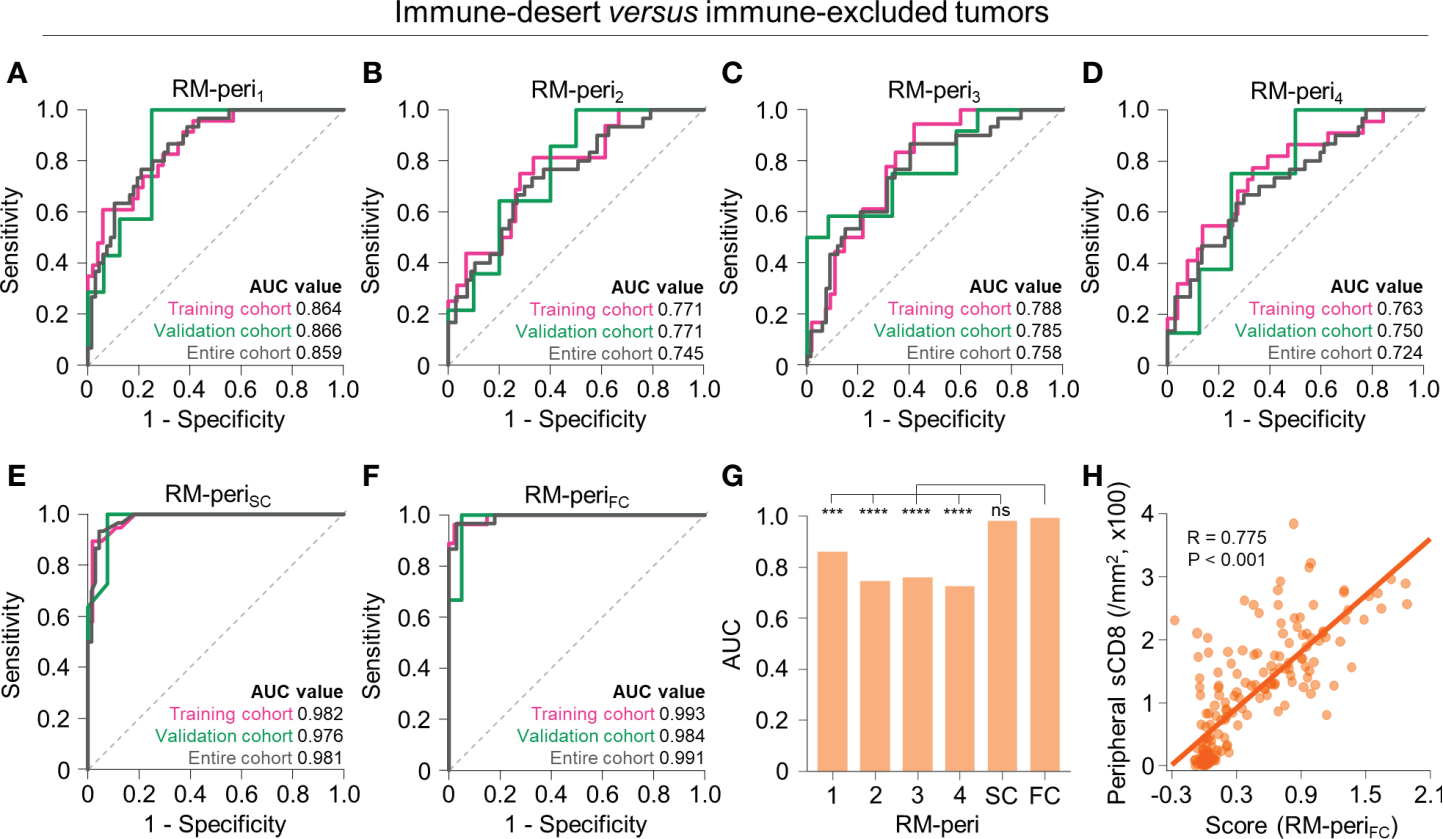
Radiomic models to predict immune-desert *versus* excluded immunophenotypes on the basis of radiomic features from the peripheral tumor. **(A–F)** The receiver operating characteristic curves of RM-peri_1_ **(A)**, RM-peri_2_ **(B)**, RM-peri_3_ **(C)**, RM-peri_4_ **(D)**, RM-peri_SC_ **(E)**, and RM-peri_FC_ **(F)** for discriminating between immune-desert and excluded tumors in the training (*pink*), validation (*green*), and entire cohorts (*gray*). **(G)** The comparison of the AUC values of the six radiomic models derived from the peripheral tumor. **(H)** The correlation between the radiomic score obtained from RM-peri_SC_ and the peripheral stromal CD8^+^ T cell density. Statistical analyses were performed using Delong’s test **(G)** and Pearson’s correlation test **(H)**. ^***^ *P* < 0.001, ^****^ *P* < 0.0001. RM, radiomic model; AUC, area under the receiver operating characteristic curve; ns, not significant; sCD8, stromal CD8^+^ T cells.

The two non-inflamed immunophenotypes, immune-desert and excluded phenotypes, differ in the extent of CD8^+^ T cell infiltration in the tumor periphery. Therefore, we developed models to further discriminate the two immunophenotypes using the radiomic features obtained from the tumor periphery. The models with single RFGs selected 2 to 13 radiomic features (**Supplementary Table 6**), with the highest AUC value in RM-peri_1_ (training, AUC = 0.864; validation, AUC = 0.866) and the lowest AUC value in RM-peri_4_ (training, AUC = 0.763; validation, AUC = 0.750) (**Figures 4A–D**). The score-based combination (RM-peri_SC_, training, AUC = 0.982; validation, AUC = 0.976) and feature-based combination (RM-peri_FC_, training, AUC = 0.993; validation, AUC = 0.984) resulted in excellent performance (**Figures 4E, F**; **Supplementary Table 7**). In the entire upfront surgery cohort, the RM-peri_FC_ showed a significantly greater AUC value than models built from single RFGs (*P* < 0.05 for all comparisons), but without significant difference observed in comparison with RM-peri_SC_ (**Figure 4G**). The stromal CD8^+^ cell density at the tumor periphery positively correlated with the score obtained from RM-peri_FC_ (*P* < 0.001; **Figure 4H**).

### Combination of the models to predict the immunophenotype

**Figure 5:**
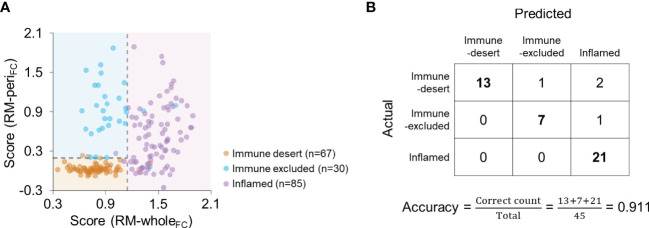
Combination of RM-whole_FC_ and RM-peri_FC_ to predict the immunophenotype. **(A)** The scores obtained from RM-whole_FC_ and RM-peri_FC_ according to the immunophenotype. The dashed lines indicate the optimal cutoff for each RM. **(B)** Confusion matrix for the combination of RM-whole_FC_ and RM-peri_FC_ in the validation cohort.

Because the feature-based combination yielded the best performance in both modeling types, we subsequently used RM-whole_FC_ and RM-peri_FC_ to predict the immunophenotype. By applying the optimal cutoff, scores from both models discriminated the immunophenotype of each patient (**Figure 5A**). In the validation cohort, the accuracy was 0.911 (**Figure 5B**), and the average F1 score was 0.950. The measurements of model performance are listed in **Supplementary Table 8**.

### Association between the models and clinicopathological features

Next, we explored the association between radiomic scores from the models and clinicopathological features. As expected, the score from RM-whole_FC_ was significantly higher in HER-2 enriched and basal-like subtypes than in luminal tumors (**Supplementary Figure 5A**). The score from RM-peri_FC_ was significantly lower in the luminal A subtype than in other subtypes (**Supplementary Figure 5B**). Regarding the close relationship between the immunophenotype and molecular subtype, we evaluated the performance of the models for each single molecular subtype. Both RM-whole_FC_ and RM-peri_FC_ displayed excellent performances with AUC values close to 1, except for RM-whole_FC_ in the basal-like subtype (AUC = 0.867; **Supplementary Figures 5C, D**). No other parameter, including pathologic T/N stages, histologic grade, vascular invasion, and lymphatic invasion, was associated with the scores from RMs, except for the positive association between the histologic grade and the score from RM-peri_FC_ (**Supplementary Figure 6A, B**).

### Association between the models and response to NACT

**Figure 6:**
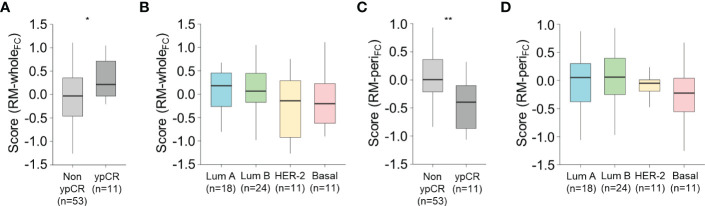
Association between the score from the radiomic models and the response to neoadjuvant chemotherapy. **(A, B)** The score obtained from RM-whole_FC_ according to the pathologic response to neoadjuvant chemotherapy **(A)** and molecular subtype **(B)**. **(C, D)** The score obtained from RM-whole_FC_ according to the pathologic response to neoadjuvant chemotherapy **(C)** and molecular subtype **(D)**. Statistical analyses were performed using Student’s *t*-test **(A, C)** and one-way ANOVA with *post-hoc* Tukey’s test **(B, D)**. ^*^ *P* < 0.05, ^**^ *P* < 0.01. ypCR, pathologic complete response.

Since a higher level of CD8^+^ TIL infiltration is reportedly associated with better response to NACT (23, 24), we tested our models to predict the response to NACT in a discrete cohort (n = 64). The pre-treatment score from RM-whole_FC_, which positively correlated with the stromal density of CD8^+^ TILs at the central tumor (as mentioned before; see **Figure 3H**), was significantly higher in patients with ypCR than in those with non-ypCR (*P* = 0.015, Student’s *t*-test; **Figure 6A**), which was consistent with previously reported results. Notably, the score did not differ according to the molecular subtype (one-way ANOVA, *P* = 0.24; **Figure 6B**), suggesting that a higher score in the ypCR group might not be because of the predominance of NACT-sensitive subtypes in that group. Intriguingly, we also found that the pre-treatment score from RM-peri_FC_ positively correlated with the stromal density of CD8^+^ T cells at the tumor periphery (as mentioned before; see **Figure 4H**), which was higher in the non-ypCR group than in the ypCR group (*P* = 0.006, Student’s t-test; **Figure 6C**), without significantly differing according to the molecular subtype (one-way ANOVA, *P* = 0.68; **Figure 6D**). This result suggests that the potential underlying mechanisms of immune-exclusion may be associated with resistance to NACT. Collectively, the use of our RMs was verified in the different treatment settings.

## Discussion

Despite accumulating evidence supporting the importance of immunophenotype in breast cancer, non-surgical methods are still unavailable to predict the immunophenotype. Here, we developed RMs to discriminate the immune-desert, excluded, and inflamed phenotypes of breast cancer. The models showed excellent performances in the cohort of patients undergoing upfront surgery, and their clinical use was also verified in association with response to NACT. To the best of our knowledge, this is the first study to build RMs predicting CD8^+^ T cell infiltration and its spatial distribution, regardless of the cancer type.

Several RMs based on MRI or mammography have been reported to predict the infiltration of stromal TILs in breast cancer (17–19, 25). Our data suggest that the immune-excluded tumors showed worse survival outcomes than tumors with other immunophenotypes, despite the similar level of stromal TILs between immune-excluded and inflamed tumors. The immune-excluded phenotype is characterized by the accumulation of T cells at the tumor periphery but not inside the central portion, which is associated with the immunosuppressive tumor microenvironment formed due to mechanical and functional barriers between the immune and cancer cells (26). Regarding the non-uniform properties of immune infiltrates, estimating stromal TILs alone may not provide sufficient information about the immune contexture. Despite the clinical implications of tumor immunophenotype (27), the use of RMs in differentiating immunophenotypes has not been thoroughly investigated. Accordingly, we suggest that our RMs predicting the individual immunophenotype may be clinically useful to decipher the underlying tumor-immune microenvironment in patients with breast cancer.

RMs to predict outcomes considering the spatial heterogeneity of tumors are lacking (28). Texture-based radiomic features represent the spatial arrangement of gray level pixel values; therefore, these features might be closely associated with spatial characteristics reflecting intratumoral heterogeneity. To select the corresponding ROIs, we separately developed the RMs for the overall and peripheral infiltration of CD8^+^ T cells. As a result, the models showed excellent performances for predicting the pathological characteristics represented by the spatial distribution of CD8^+^ T cells. Our methodology can also be used in future investigations to develop RMs to predict the spatial distribution of other immune cells and immune-related markers that have not been examined in the current analysis.

Our results demonstrated dissimilar performances of the RMs built from a single phase of DCE-MRI. Most radiomics studies for breast cancer have utilized the strongest enhanced phase to build the models (29–31). Tang et al. reported that the delayed phase of DCE-MRI provided better information regarding the amount of TIL infiltration (17); in contrast, our findings suggested that RMs from the early phase showed the highest AUC values in predicting both central and peripheral CD8^+^ T cell density. Additionally, combining the radiomic features from all phases significantly improved the performance compared to using features from a single phase. Therefore, this study indicates that features from the delayed phases also contain non-redundant information regarding the distribution of CD8^+^ T cells.

Tumor-infiltrating CD8^+^ T cells exhibit anti-tumor immune responses in breast cancer (32). Hence, the non-invasive prediction of CD8^+^ T cell distribution may be useful in real-world clinics in the era of immunotherapy in combination with radiotherapy and/or chemotherapy. For breast cancer, immune checkpoint inhibitors have shown improved clinical outcomes, especially in the basal-like subtype (33–35); an increased baseline density of CD8^+^ T cells is associated with a favorable response to immunotherapy (36). In a phase III clinical trial, the benefit of PD-L1 blockade in addition to chemotherapy was observed only in tumors highly infiltrated with CD8^+^ T cells (37). A recent single-cell RNA-sequencing analysis showed that the abundance and expansion of tumor-infiltrating CD8^+^ T cells with exhausted features were associated with a better response to immune checkpoint inhibitors in the basal-like subtype of breast cancer (38). Additionally, the spatial distribution of CD8^+^ T cells also appears to be an important predictive factor for using immunotherapy for breast cancer (13). The mechanisms of immune escape and resistance to PD-1/PD-L1 inhibition involve immune-desert and excluded tumors, whereas inflamed tumors exhibit favorable therapeutic responses (26). Therefore, our RMs discriminating the different immunophenotypes may be applied for stratifying patients treated with immune checkpoint blockade.

When we applied the RMs in a discrete NACT cohort, the scores remarkably differed according to treatment responses. Several RMs have been developed to predict response to NACT in breast cancer (30, 39–43); however, the biological backgrounds of the models have not been studied well. In line with the reported positive correlation between CD8^+^ T cell infiltration and response to NACT (23, 24), this study demonstrated the link between RMs and differential response to NACT, mediated by the infiltration of CD8^+^ T cells. Notably, our results also showed that complete responders to NACT may contain a significantly lower amount of CD8^+^ T cells at the tumor periphery. The potential association between the spatial distribution of immune cells and the pathological response to NACT needs to be elucidated in future studies.A major limitation of this study is that the subjects were from a single institution with a uniform DCE-MRI protocol. The performance of our modeling approach needs to be tested using different protocols, and an additional analysis with an independent external cohort is necessary for further validation of our models. To facilitate clinical application in future studies, a more spatially oriented analysis of the tumor and lymphocyte populations will be helpful. More automated approaches to analyze radiomic features and outcomes are also needed. Nevertheless, in this study, the selected radiomic features were highly stable to interobserver variability, and both radiomic features and immunophenotype information were considered reproducible. To expand the current point of view, combining pathological and radiomic features may be useful for predicting pCR. Regarding different biological characteristics and tumor aggressiveness according to molecular subtypes, the predictability of our models needs to be further validated for each subset. Despite these things listed above, this is the first study to establish a non-invasive imaging tool to decipher the tumor-immune microenvironment, reflecting the heterogenous contexture of immune cells within tumor tissues.

In summary, we developed DCE-MRI-based RMs to predict the individual immunophenotype of breast cancer based on the spatial distribution of CD8^+^ T cells. The models showed excellent performance, which was associated with a favorable response to NACT. In the contemporary era of cancer immunotherapy, our proposed models can be used to stratify patients non-invasively based on the status of the tumor-immune microenvironment. Large-scale studies are needed to further validate our models.

## Data availability statement

The original contributions presented in the study are included in the article/**Supplementary Material**. Further inquiries can be directed to the corresponding author.

## Ethics statement

The studies involving human participants were reviewed and approved by Institutional Review Board of Kyung Hee University Hospital. The ethics committee waived the requirement of written informed consent for participation.

## Author contributions

Conceptualization: SJ, S-WK, and YL. Methodology: SJ and S-WK. Formal analysis: SJ, S-WK, and YL. Investigation: SJ, S-WK, KN, MS, Y-MS, and YL. Resources: YL. Data curation: S-WK, KN, MS, and Y-MS. Writing: SJ, S-WK, and YL. Supervision: YL. Funding acquisition: YL. All authors contributed to the article and approved the submitted version.
