# Supplementary material for: Radiomic models based on magnetic resonance imaging predict the spatial distribution of CD8+ tumor-infiltrating lymphocytes in breast cancer
Source: Front Immunol. 2022 Dec 19;13:1080048. doi: 10.3389/fimmu.2022.1080048 (PMC9806253; doi:10.3389/fimmu.2022.1080048)
Supplement: Supplementary file 1 [file DataSheet_1.docx]

**Supplementary Methods**

**Extracted radiomics features**

**1. Geometry**

N_V_ is the number of isotropically resampled voxels (1 × 1 × 1 mm^3^) of the whole tumor volume.

1.1 Volume

$$volume=N_{V} ({mm}^{3})$$

**2. First-order features**

First order features describe the distribution of gray values within a region of interest. The number of voxels in given ROI is denoted with ***N***. The probability vector of the first order histogram with ***N_g_*** (=64) discrete bins is denoted with ***P***.

2.1. Mean

$$mean=u=\sum_{i=1}^{N_{g}} i \times P(i)$$

2.2. Variance

$$variance=\sigma^{2}=\sum_{i=1}^{N_{g}} {(i-u)}^{2}\times P(i)$$

2.3. Interquartile range

$$interquartile range=P_{75\%}-P_{25\%}$$

2.4. Energy

$$energy=\sum_{i=1}^{N_{g}} \left( N\times P\left( i \right) \right)^{2}$$

2.5. Entropy

$$entropy=-\sum_{i=1}^{N_{g}} \log_{2} P(i)\times P(i)$$

2.6. Uniformity

$$uniformity=\sum_{i=1}^{N_{g}} {P(i)}^{2}$$

2.7. Skewness

$$skewness=\frac{\sum_{i=1}^{N_{g}} {(i-u)}^{3}\times P(i)}{\sigma^{3}}$$

2.8. Kurtosis

$$kurtosis= \frac{\sum_{i=1}^{N_{g}} {(i-u)}^{4}\times P(i)}{\sigma^{4}}-3$$

**3. Gray-level co-occurrence matrix (GLCM) based texture features**

A GLCM of size N_g_ × N_g_ (=64 × 64) is denoted with ***P*** and describes textural information of given ROI. The **P**(*i*, *j*) is defined as the number of times a voxels of intensity *i* and the voxel in a distance δ of intensity j. The distance vector δ is from $\left\{ \left( x,y,z \right)|x\in\left( -1,0,1 \right) and y\in\left( -1,0,1 \right) and z\in\left( -1,0,1 \right) and (x,y,z)\neq(0,0,0) \right\}$. In case of 2D features, δ is from $\left\{ \left( x,y \right)|x\in\left( -1,0,1 \right) and y\in\left( -1,0,1 \right) and (x,y)\neq(0,0) \right\}$. To account for discretization length differences, neighbors at a distance of 1, $\sqrt{2}$, and $\sqrt{3}$ were given a weight of 1, $1/\sqrt{2}$, and $1/\sqrt{3}$, respectively.

• p(i,j) = ${P(i,j)}/{\sum P(i,j)}$

• μ = mean of p(i,j)

• μ_x_ = $\sum_{i}^{N_{g}} \sum_{j}^{N_{g}} p\left( i,j \right)i$

• μ_y_ = $\sum_{i}^{N_{g}} \sum_{j}^{N_{g}} p\left( i,j \right)j$

• σ = standard deviation of p(i,j)

• p_x+y_(k) = $\sum_{i}^{N_{g}} \sum_{j}^{N_{g}} p\left( i,j \right)$, $i+j=k$

• p_x-y_(k) = $\sum_{i}^{N_{g}} \sum_{j}^{N_{g}} p\left( i,j \right)$, $\left| i-j \right|=k$

• HX = $-\sum_{i}^{N_{g}} p_{x}\left( i \right)\log_{2} \left( p_{x}\left( i \right)+\epsilon\right)$, where $p_{x}(i)=\sum_{j}^{N_{g}} p(i,j)$

• HY = $-\sum_{j}^{N_{g}} p_{y}\left( j \right)\log_{2} \left( p_{y}\left( j \right)+\epsilon\right)$, where $p_{y}(j)=\sum_{i}^{N_{g}} p(i,j)$

• HXY = $-\sum_{i}^{N_{g}} \sum_{j}^{N_{g}} p(i,j)\log_{2} \left( p\left( i,j \right)+\epsilon\right)$

• HXY1 = $-\sum_{i}^{N_{g}} \sum_{j}^{N_{g}} p(i,j)\log_{2} \left( p_{x}(i)p_{y}(j)+\epsilon\right)$

• HXY2 = $-\sum_{i}^{N_{g}} \sum_{j}^{N_{g}} {p_{x}(i)p_{y}(j)\log}_{2} \left( p_{x}(i)p_{y}(j)+\epsilon\right)$

• P_o_ = $\sum_{i}^{N_{g}} p\left( i,i \right)$

• P_e_ = $\sum_{i}^{N_{g}} p\left( i,: \right)p(:,i)$

3.1. Energy

$$energy=\sum_{i=1}^{N_{g}} \sum_{j=1}^{N_{g}} {p(i,j)}^{2}$$

3.2. Contrast

$$contrast=\sum_{i=1}^{N_{g}} \sum_{j=1}^{N_{g}} \left( i-j \right)^{2}p(i,j)$$

3.3. Entropy

$$entropy=-\sum_{i=1}^{N_{g}} \sum_{j=1}^{N_{g}} \log_{2} \left[ P\left( i,j \right) \right]\times p(i,j)$$

3.4. Homogeneity 1

$$homogeneity 1=\sum_{i=1}^{N_{g}} \sum_{j=1}^{N_{g}} \frac{p(i,j)}{1+\left| i-j \right|}$$

3.5. Homogeneity 2

$$homogeneity 2=\sum_{i=1}^{N_{g}} \sum_{j=1}^{N_{g}} \frac{p(i,j)}{1+\left| i-j \right|^{2}}$$

3.6. Correlation

$$correlation=\frac{1}{\sigma}\sum_{i=1}^{N_{g}} \sum_{j=1}^{N_{g}} (i-\mu)(j-\mu)p(i,j)$$

3.7. Variance 1

$$variance 1=\frac{1}{{2N_{g}}^{2}}\sum_{i=1}^{N_{g}} \sum_{j=1}^{N_{g}} \left[ \left( i-\mu\right)^{2}p\left( i,j \right)+\left( j-\mu\right)^{2}p(i,j) \right]$$

3.8. Variance 2

$$variance 2=\sum_{i=1}^{N_{g}} \sum_{j=1}^{N_{g}} {(i-\mu)}^{2}p(i,j)$$

3.9. Sum Average 1

$$sum average 1=\frac{1}{{2N_{g}}^{2}}\sum_{i=1}^{N_{g}} \sum_{j=1}^{N_{g}} \left( i+j \right)p(i,j)$$

3.10. Sum Average 2

$$sum average 2=\sum_{i=2}^{2N_{g}} ip_{x+y}(i)$$

3.11. Dissimilarity

$$dissimilarity=\sum_{i=1}^{N_{g}} \sum_{j=1}^{N_{g}} \left| i-j \right|p(i,j)$$

3.12. Autocorrelation

$$autocorrelation=\sum_{i=1}^{N_{g}} \sum_{j=1}^{N_{g}} i\times j\times p(i,j)$$

3.13. Difference Entropy

$$difference entropy=-\sum_{i=0}^{N_{g}-1} \log_{2} {[p}_{x-y}(i)]\times p_{x-y}(i)$$

3.14. Sum Entropy

$$sum entropy=-\sum_{i=2}^{2N_{g}} \log_{2} {[p}_{x+y}(i)]\times p_{x+y}(i)$$

3.15. Sum Variance (where SE is sum entropy)

$$sum variance=\sum_{i=2}^{{2N}_{g}} {(i-SE)}^{2} p_{x+y}(i)$$

3.16. Cluster Prominence

$$cluster prominence=\sum_{i=1}^{N_{g}} \sum_{j=1}^{N_{g}} \left( i+j-\mu_{x}-\mu_{y} \right)^{4}p(i,j)$$

3.17. Cluster Shade

$$cluster shade=\sum_{i=1}^{N_{g}} \sum_{j=1}^{N_{g}} \left( i+j-\mu_{x}-\mu_{y} \right)^{3}p(i,j)$$

3.18. Cluster Tendency

$$cluster tendency=\sum_{i=1}^{N_{g}} \sum_{j=1}^{N_{g}} \left( i+j-\mu_{x}-\mu_{y} \right)^{2}p(i,j)$$

3.19. Maximum Probability

$$maximum probability=max\left( p(i,j) \right)$$

3.20. Informational Measure of Correlation (IMC) 1

$$IMC 1= \frac{HXY-HXY1}{max\{HX,HY\}}$$

3.21. Informational Measure of Correlation (IMC) 2

$$IMC 2= \sqrt{1-e^{-2(HXY2-HXY)}}$$

3.22. Inverse Difference Moment (IDM)

$$IDM=\sum_{i=1}^{N_{g}} \sum_{j=1}^{N_{g}} \frac{p(i,j)}{{(i-j)}^{2}} (i\neq j)$$

3.23. Inverse Difference Moment Normalized (IDMN)

$$IDMN=\sum_{i=1}^{N_{g}} \sum_{j=1}^{N_{g}} \frac{p(i,j)}{1+\left( \frac{i-j}{N_{g}} \right)^{2}}$$

3.24. Inverse Difference Normalized (IDN)

$$IDN=\sum_{i=1}^{N_{g}} \sum_{j=1}^{N_{g}} \frac{p(i,j)}{1+\frac{\left| i-j \right|}{N_{g}}}$$

3.25. Agreement

$$agreement= \frac{P_{o}-P_{e}}{1-P_{e}}$$

**4. Gray-level run-length matrix (GLRLM) based texture features**

A GLRLM of size N_g_ (=64) × N_r_ is denoted with ***P*** , where N_r_ is the number of different run lengths, and describes textural information of the given ROI. The **P**(*i*, *j*) is defined as the number of runs with *j* consecutive voxels of intensity *i* with direction of δ. The distance vector δ is from $\left\{ \left( x,y,z \right)|x\in\left( -1,0,1 \right) and y\in\left( -1,0,1 \right) and z\in\left( -1,0,1 \right) and (x,y,z)\neq(0,0,0) \right\}$. In case of 2D features, δ is from $\left\{ \left( x,y \right)|x\in\left( -1,0,1 \right) and y\in\left( -1,0,1 \right) and (x,y)\neq(0,0) \right\}$. To account for discretization length differences, neighbors at a distance of 1, $\sqrt{2}$, and $\sqrt{3}$ were given a weight of 1, $1/\sqrt{2}$, and $1/\sqrt{3}$, respectively.

• p(i,j) = ${P(i,j)}/{\sum P(i,j)}$

• μ_x_ = mean of p_x_(i)

• μ_y_ = mean of p_y_(j)

• N = number of voxels

4.1. Short Run Emphasis

$$small run emphasis=\sum_{i=1}^{N_{g}} \sum_{j=1}^{N_{r}} \frac{p(i,j)}{j^{2}}$$

4.2. Long Run Emphasis

$$long run emphasis=\sum_{i=1}^{N_{g}} \sum_{j=1}^{N_{r}} j^{2}p(i,j)$$

4.3. Gray-Level Nonuniformity

$$gray level nonuniformity=\sum_{i=1}^{N_{g}} \left( \sum_{j=1}^{N_{r}} p(i,j) \right)^{2}$$

4.4. Run-Length Nonuniformity

$$run length nonuniformity=\sum_{j=1}^{N_{r}} \left( \sum_{i=1}^{N_{g}} p(i,j) \right)^{2}$$

4.5. Run Percentage

$$run percentage=\frac{1}{N}\sum_{i=1}^{N_{g}} \sum_{j=1}^{N_{r}} P(i,j)$$

4.6. Low Gray-Level Run Emphasis

$$low gray level run emphasis=\sum_{i=1}^{N_{g}} \sum_{j=1}^{N_{r}} \frac{p(i,j)}{i^{2}}$$

4.7. High Gray-Level Run Emphasis

$$high gray level run emphasis=\sum_{i=1}^{N_{g}} \sum_{j=1}^{N_{r}} i^{2}p(i,j)$$

4.8. Short Run Low Gray-Level Emphasis

$$short run low gray level emphasis=\sum_{i=1}^{N_{g}} \sum_{j=1}^{N_{r}} \frac{p(i,j)}{i^{2}j^{2}}$$

4.9. Short Run High Gray Level Emphasis

$$short run high gray level emphasis=\sum_{i=1}^{N_{g}} \sum_{j=1}^{N_{r}} \frac{i^{2}p(i,j)}{j^{2}}$$

4.10. Long Run Low Gray-Level Emphasis

$$long run low gray level emphasis=\sum_{i=1}^{N_{g}} \sum_{j=1}^{N_{r}} i^{2}j^{2}p(i,j)$$

$$long run low gray level emphasis=\sum_{i=1}^{N_{g}} \sum_{j=1}^{N_{r}} \frac{j^{2}p(i,j)}{i^{2}}$$

4.11. Long Run High Gray-Level Emphasis

$$long run high gray level emphasis=\sum_{i=1}^{N_{g}} \sum_{j=1}^{N_{r}} i^{2}j^{2}p(i,j)$$

4.12. Gray-Level Variance

$$gray level variane=\sum_{i=1}^{N_{g}} \sum_{j=1}^{N_{r}} {(i-\mu_{x})}^{2}p(i,j)$$

4.13. Run-Length Variance

$$run length variane=\sum_{i=1}^{N_{g}} \sum_{j=1}^{N_{r}} {(j-\mu_{y})}^{2}p(i,j)$$

**5. Gray-level size-zone matrix (GLSZM) based texture features**

A GLSZM of size N_g_ (=64) × N_z_ is denoted with ***P***, where N_z_ is the number of different zone sizes, and describes textural information of the given ROI. A gray level zone is defined as the number of connected voxels with the same intensity. Voxels are considered connected if the distance between the two voxels is δ. The **P**(*i*, *j*) is defined as the number of zones with size of *j* and intensity of *i*. The distance vector δ is from $\left\{ \left( x,y,z \right)|x\in\left( -1,0,1 \right) and y\in\left( -1,0,1 \right) and z\in\left( -1,0,1 \right) and (x,y,z)\neq(0,0,0) \right\}$. In case of 2D features, δ is from $\left\{ \left( x,y \right)|x\in\left( -1,0,1 \right) and y\in\left( -1,0,1 \right) and (x,y)\neq(0,0) \right\}$. To account for discretization length differences, neighbors at a distance of 1, $\sqrt{2}$, and $\sqrt{3}$ were given a weight of 1, $1/\sqrt{2}$, and $1/\sqrt{3}$, respectively.

• p(i,j) = ${P(i,j)}/{\sum P(i,j)}$

• μ_x_ = mean of p_x_(i)

• μ_y_ = mean of p_y_(j)

• N = number of voxels

5.1. Small Zone Emphasis

$$small zone emphasis=\sum_{i=1}^{N_{g}} \sum_{j=1}^{N_{z}} \frac{p(i,j)}{j^{2}}$$

5.2. Large Zone Emphasis

$$large zone emphasis=\sum_{i=1}^{N_{g}} \sum_{j=1}^{N_{z}} j^{2}p(i,j)$$

5.3. Gray-Level Nonuniformity

$$gray level nonuniformity=\sum_{i=1}^{N_{g}} \left( \sum_{j=1}^{N_{z}} p(i,j) \right)^{2}$$

5.4. Zone-Size Nonuniformity

$$zone size nonuniformity=\sum_{j=1}^{N_{z}} \left( \sum_{i=1}^{N_{g}} p(i,j) \right)^{2}$$

5.5. Zone Percentage

$$zone percentage=\frac{1}{N}\sum_{i=1}^{N_{g}} \sum_{j=1}^{N_{z}} P(i,j)$$

5.6. Low Gray-Level Zone Emphasis

$$low gray level zone emphasis=\sum_{i=1}^{N_{g}} \sum_{j=1}^{N_{z}} \frac{p(i,j)}{i^{2}}$$

5.7. High Gray-Level Zone Emphasis

$$high gray level zone emphasis=\sum_{i=1}^{N_{g}} \sum_{j=1}^{N_{z}} i^{2}p(i,j)$$

5.8. Small Zone Low Gray-Level Emphasis

$$small zone low gray level emphasis=\sum_{i=1}^{N_{g}} \sum_{j=1}^{N_{z}} \frac{p(i,j)}{i^{2}j^{2}}$$

5.9. Small Zone High Gray-Level Emphasis

$$small zone high gray level emphasis=\sum_{i=1}^{N_{g}} \sum_{j=1}^{N_{z}} \frac{i^{2}p(i,j)}{j^{2}}$$

5.10. Large Zone Low Gray-Level Emphasis

$$large zone low gray level emphasis=\sum_{i=1}^{N_{g}} \sum_{j=1}^{N_{z}} \frac{j^{2}p(i,j)}{i^{2}}$$

5.11. Large Zone High Gray-Level Emphasis

$$large zone high gray level emphasis=\sum_{i=1}^{N_{g}} \sum_{j=1}^{N_{z}} i^{2}j^{2}p(i,j)$$

5.12. Gray-Level Variance

$$gray level variance=\sum_{i=1}^{N_{g}} \sum_{j=1}^{N_{z}} {(i-\mu_{x})}^{2}p(i,j)$$

5.13. Zone-Size Variance

$$zone size variane=\sum_{i=1}^{N_{g}} \sum_{j=1}^{N_{z}} {(j-\mu_{y})}^{2}p(i,j)$$

**6. Neighborhood gray-tone difference matrix (NGTDM) based texture features**

A NGTDM of size N_g_ (=64) × 1 is denoted with ***P***. The P(i) is defined as $\sum_{all voxels\in\{N_{i}\}} \left| i-\bar{A_{i}} \right|$ if $N_{i}>0$ and 0 if $N_{i}=0$, where {N_i_} is the set of all voxels with gray-level *i*, N_i_ is the number of voxels with gray-level *i*, and $\bar{A_{i}}$ is the average gray-level of the connected neighbors around voxels with intensity *i*. Voxels are considered connected if the distance between the two voxels is δ. The **P**(*i*, *j*) is defined as the number of zones with size of *j* and intensity of *i*. The distance vector δ is from $\left\{ \left( x,y,z \right)|x\in\left( -1,0,1 \right) and y\in\left( -1,0,1 \right) and z\in\left( -1,0,1 \right) and (x,y,z)\neq(0,0,0) \right\}$. In case of 2D features, δ is from $\left\{ \left( x,y \right)|x\in\left( -1,0,1 \right) and y\in\left( -1,0,1 \right) and (x,y)\neq(0,0) \right\}$. To account for discretization length differences, neighbors at a distance of 1, $\sqrt{2}$, and $\sqrt{3}$ were given a weight of 1, $1/\sqrt{2}$, and $1/\sqrt{3}$, respectively.

• N = number of voxels

• N_e_ = effective number of gray-levels

•$\epsilon$ = small number to prevent infinite

6.1. Coarseness

$$coarseness=\frac{N}{\epsilon+\sum_{i=1}^{N_{g}} N_{i}P(i)}$$

6.2. Contrast

$$contrast=\left[ \frac{1}{N^{3}}\sum_{i=1}^{N_{g}} P(i) \right]\left[ \frac{1}{N_{e}(N_{e}-1)}\sum_{i=1}^{N_{g}} \sum_{j=1}^{N_{g}} N_{i}N_{j}{(i-j)}^{2} \right]$$

6.3. Busyness

$$busyness=\frac{\sum_{i=1}^{N_{g}} N_{i}P(i)}{\sum_{i=1}^{N_{g}} \sum_{j=1}^{N_{g}} (iN_{i}-jN_{j})}, N_{i}\neq0, N_{j}\neq0$$

6.4. Complexity

$$complexity=\sum_{i=1}^{N_{g}} \sum_{j=1}^{N_{g}} \frac{\left| i-j \right|\left[ N_{i}P\left( i \right)+N_{j}P(j) \right]}{N(N_{i}+N_{j})}, N_{i}\neq0, N_{j}\neq0$$

6.5. Strength

$$strength=\frac{\sum_{i=1}^{N_{g}} \sum_{j=1}^{N_{g}} (N_{i}+N_{j}){(i-j)}^{2}}{N\times\left[ \epsilon+\sum_{i=1}^{N_{g}} P(i) \right]}, N_{i}\neq0, N_{j}\neq0$$
