# Supplementary material for: Radiomic models based on magnetic resonance imaging predict the spatial distribution of CD8+ tumor-infiltrating lymphocytes in breast cancer
Source: Front Immunol. 2022 Dec 19;13:1080048. doi: 10.3389/fimmu.2022.1080048 (PMC9806253; doi:10.3389/fimmu.2022.1080048)
Supplement: Supplementary file 2 [file DataSheet_2.pdf]

Supplementary Figure 1

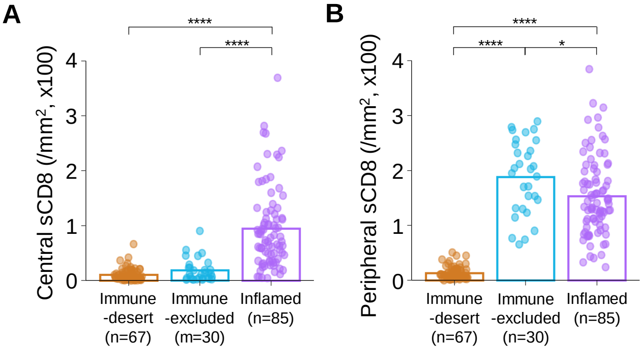

Supplementary Figure 1. Density of stromal CD8^{+} T cells according to the immunophenotype. (A–B) Density of central (A) and peripheral (B) CD8^{+} T cells in each immunophenotype. Statistical analysis was performed using one-way ANOVA with post-hoc Tukey's test. *P < 0.05, ****P < 0.0001.

Supplementary Figure 2

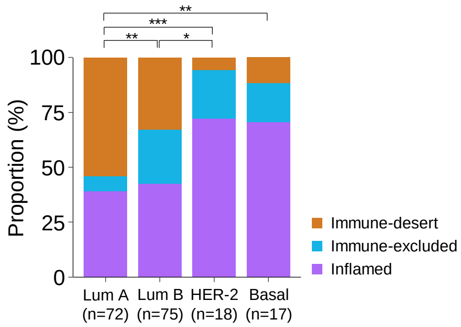

Supplementary Figure 2. Distribution of immunophenotypes according to the molecular subtype. Statistical analysis was performed using Chi-squared test. *P < 0.05, **P < 0.01, ***P < 0.001.

Supplementary Figure 3

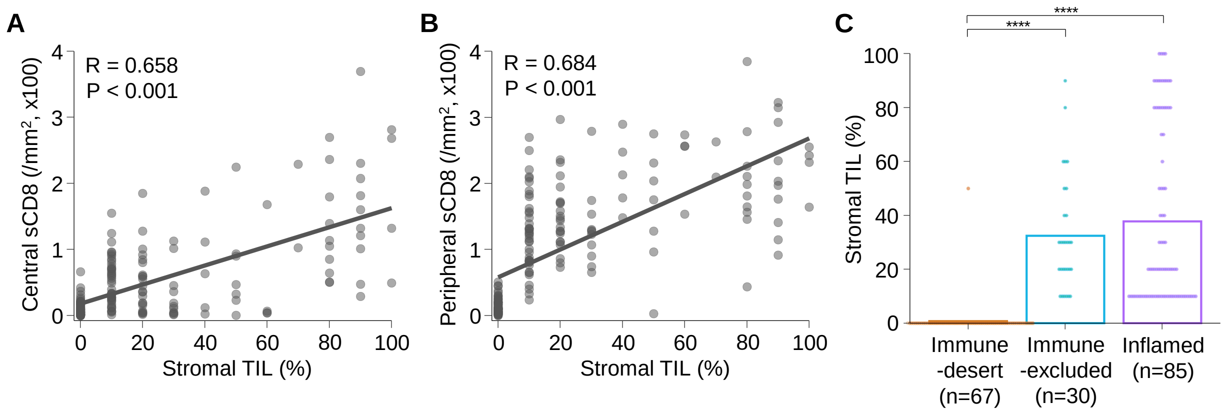

Supplementary Figure 3. Association between stromal TIL and distribution of CD8$^+$ T cells. (A–B) Correlation between stromal TIL infiltration and density of stromal CD8$^+$ T cells at center (A) and periphery of tumor (B). (C) Stromal TIL infiltration in each immunophenotype. Statistical analyses were performed using Pearson's correlation test (A–B) and one-way ANOVA with post-hoc Tukey's test. ****P < 0.0001. TIL, tumor-infiltrating lymphocyte.

Supplementary Figure 4

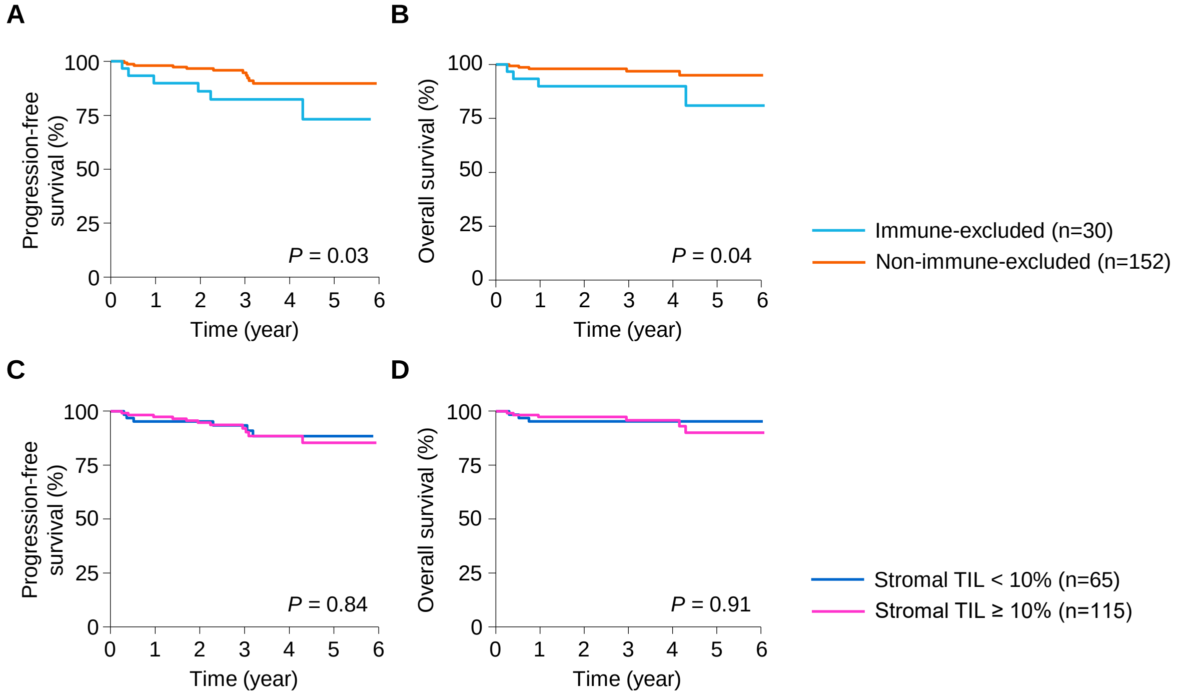

Supplementary Figure 4. Immune cell infiltration and survival outcomes. (A–B) Kaplan-Meier curves of progression-free survival (A) and overall survival (B) in immune-excluded and non-immune-excluded tumors. (C–D) Kaplan-Meier curves of progression-free survival (C) and overall survival (D) in tumors with stromal TIL <10% and ≥ 10%. Statistical analysis was performed using Cox proportional hazard model. TIL, tumor-infiltrating lymphocyte.

Supplementary Figure 5

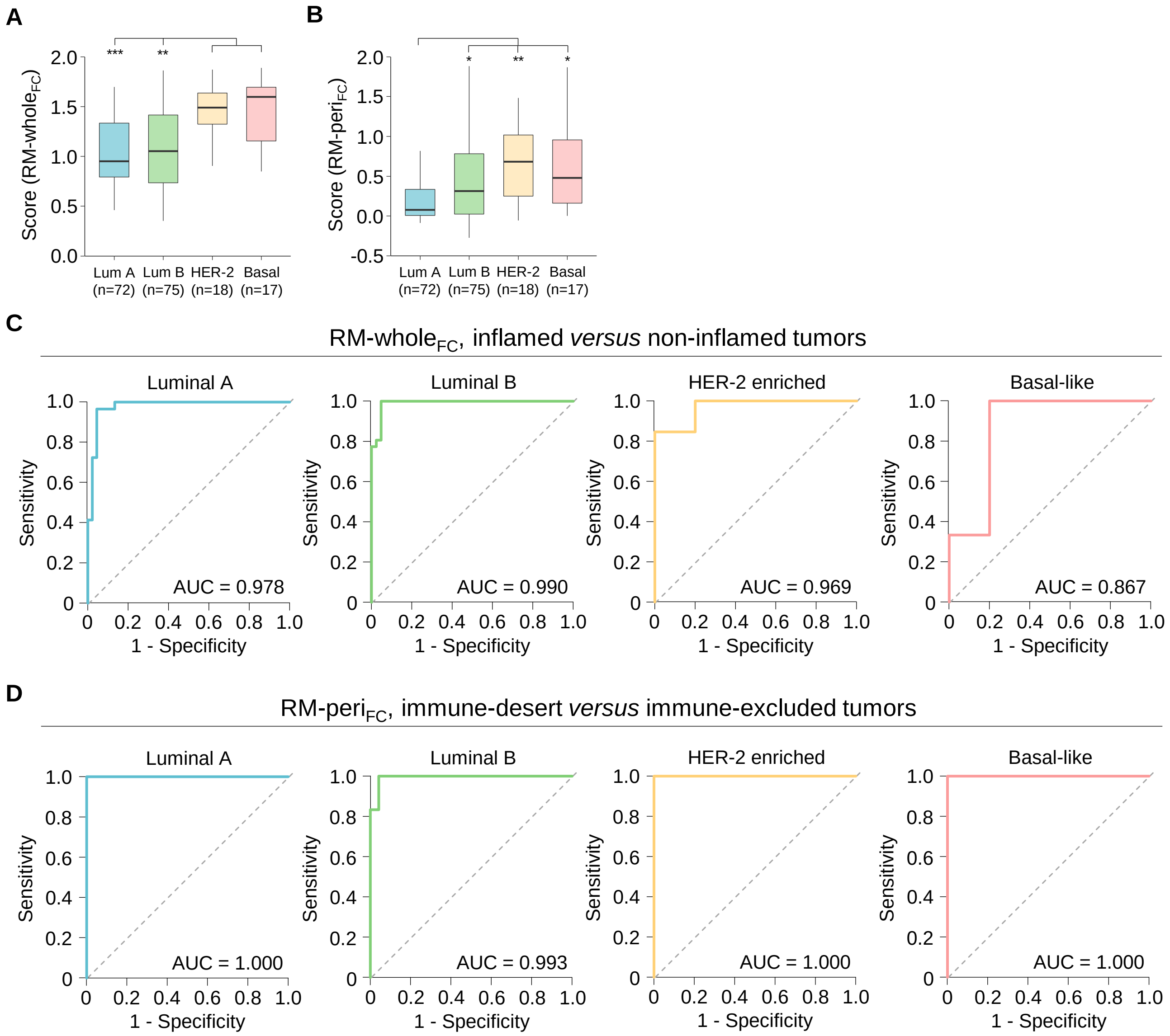

Supplementary Figure 5. Performance of the radiomic models according to molecular subtype. (A–B) The scores from RM-whole$_{FC}$ (A) and RM-peri$_{FC}$ (B) in each molecular subtype. (C–D) The ROC curves of RM-whole$_{FC}$ and RM-peri$_{FC}$ for prediction of immunophenotype in each molecular subtype. Statistical analysis was performed using one-way ANOVA with post-hoc Tukey's test. *P < 0.05, **P < 0.01, ***P < 0.001.

Supplementary Figure 6

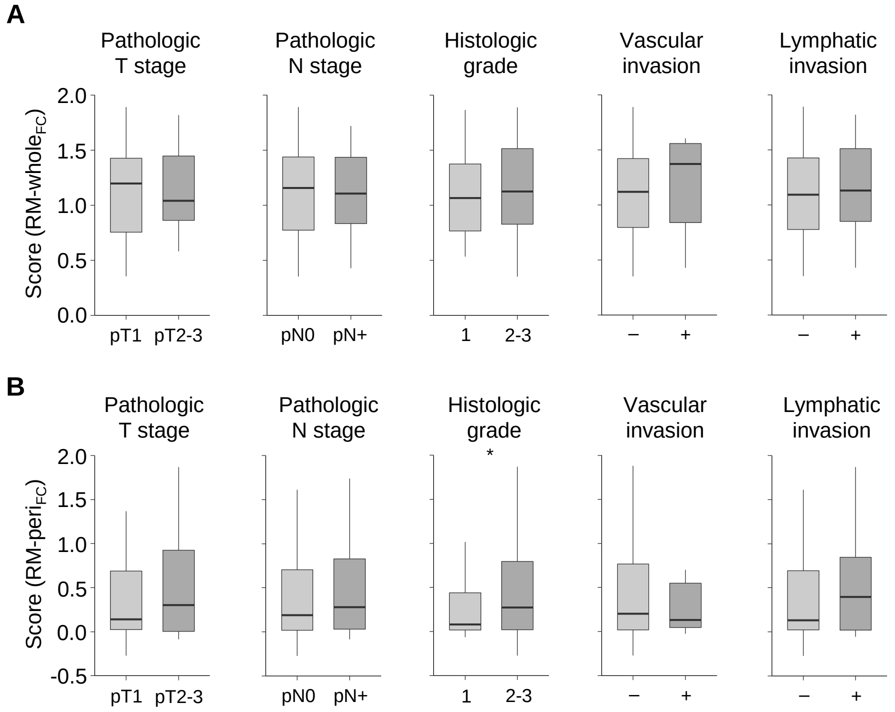

Supplementary Figure 6. Association between pathologic characteristics of tumors and radiomic models. (A–B) The scores from RM-whole$_{FC}$ (A) and RM-peri$_{FC}$ (B) according to pathologic T and N stages, histologic grade, vascular invasion, and lymphatic invasion. Statistical analysis was performed using *t*-test. *$P < 0.05$, **$P < 0.01$, ***$P < 0.001$.
