# Supplementary material for: Radiomic models based on magnetic resonance imaging predict the spatial distribution of CD8+ tumor-infiltrating lymphocytes in breast cancer
Source: Front Immunol. 2022 Dec 19;13:1080048. doi: 10.3389/fimmu.2022.1080048 (PMC9806253; doi:10.3389/fimmu.2022.1080048)
Supplement: Supplementary file 3 [file Table_1.docx]

|  | Training cohort  (n=137) | Validation cohort  (n=45) | P-value |
| --- | --- | --- | --- |
| Age (year) |  |  | 0.574 |
| Mean ± SD | 56.8 ± 10.6 | 57.9 ± 11.0 |  |
| Pathologic T stage |  |  | 0.788 |
| T1 | 87 (63.5%) | 29 (65.9%) |  |
| T2 | 44 (32.1%) | 13 (29.5%) |  |
| T3 | 6 (4.4%) | 3 (6.7%) |  |
| Pathologic N stage |  |  | 0.089 |
| N0 | 105 (76.6%) | 28 (62.2%) |  |
| N+ | 32 (23.4%) | 17 (37.8%) |  |
| Histology |  |  | 0.979 |
| Invasive ductal carcinoma | 125 (91.2%) | 41 (91.1%) |  |
| Others | 12 (8.8%) | 4 (8.9%) |  |
| Estrogen receptor |  |  | 0.546 |
| Expressed | 108 (78.8%) | 38 (84.4%) |  |
| Not expressed | 29 (21.2%) | 7 (15.6%) |  |
| Progesterone receptor |  |  | 0.315 |
| Expressed | 101 (73.7%) | 29 (64.4%) |  |
| Not expressed | 36 (26.3%) | 16 (35.6%) |  |
| Her-2 overexpression |  |  | 0.258 |
| Present | 35 (25.5%) | 7 (15.6%) |  |
| Absent | 100 (73.0%) | 38 (84.4%) |  |
| Equivocal | 2 (1.5%) | 0 (0.0%) |  |
| Subtype |  |  | 0.261 |
| Luminal A | 57 (41.6%) | 15 (33.3%) |  |
| Luminal B | 52 (38.0%) | 23 (51.1%) |  |
| Her-2 enriched | 16 (11.7%) | 2 (4.4%) |  |
| Triple-negative | 12 (8.8%) | 5 (11.1%) |  |
| Histologic grade |  |  | 0.693 |
| Grade 1 | 36 (26.3%) | 12 (26.7%) |  |
| Grade 2 | 49 (35.8%) | 20 (44.4%) |  |
| Grade 3 | 48 (35.0%) | 12 (26.7%) |  |
| Not reported | 4 (2.9%) | 1 (2.2%) |  |
| Lymphatic invasion |  |  | 0.520 |
| Present | 33 (24.1%) | 13 (28.9%) |  |
| Absent | 104 (75.9%) | 32 (71.1%) |  |
| Vascular invasion |  |  | 0.840 |
| Present | 8 (5.8%) | 3 (6.7%) |  |
| Absent | 129 (94.2%) | 42 (93.3%) |  |
| Immunophenotype |  |  | 0.958 |
| Immune desert | 51 (37.2%) | 16 (35.6%) |  |
| Immune excluded | 22 (16.1%) | 8 (17.8%) |  |
| Inflamed | 64 (46.7%) | 21 (46.7%) |  |

**Supplementary Table 1.** Baseline characteristics of the upfront surgery cohort.

SD, standard deviation.

|  | NACT cohort (n=64) |
| --- | --- |
| Age (year) |  |
| Mean ± SD | 52.8 ± 8.4 |
| Pathologic response |  |
| ypCR | 11 (17.2%) |
| Non-ypCR | 53 (82.8%) |
| Histology |  |
| Invasive ductal carcinoma | 57 (89.1%) |
| Others | 7 (10.9%) |
| Estrogen receptor |  |
| Expressed | 41 (64.1%) |
| Not expressed | 23 (33.9%) |
| Progesterone receptor |  |
| Expressed | 31 (48.4%) |
| Not expressed | 33 (51.6%) |
| Her-2 overexpression |  |
| Present | 19 (29.7%) |
| Absent | 43 (67.2%) |
| Equivocal | 2 (3.1%) |
| Subtype |  |
| Luminal A | 18 (28.1%) |
| Luminal B | 24 (37.5%) |
| Her-2 enriched | 11 (17.2%) |
| Triple-negative | 11 (17.2%) |

**Supplementary Table 2.** Baseline characteristics of the neoadjuvant chemotherapy cohort.

NACT, neoadjuvant chemotherapy; SD, standard deviation; ypCR, pathologic complete response.

|  | Immune-desert  (n=67) | Immune-excluded  (n=30) | Inflamed  (n=85) | *P*-value |
| --- | --- | --- | --- | --- |
| Pathologic T stage |  |  |  | 0.003 |
| T1 | 46 (68.7%) | 10 (33.3%) | 61 (71.8%) |  |
| T2 | 17 (25.4%) | 18 (60.0%) | 22 (25.9%) |  |
| T3 | 4 (6.0%) | 2 (6.7%) | 2 (2.4%) |  |
| Pathologic N stage |  |  |  | 0.203 |
| N0 | 50 (74.6%) | 18 (60.0%) | 65 (76.5%) |  |
| N+ | 17 (25.4%) | 12 (40.0%) | 20 (23.5%) |  |
| Histology |  |  |  | 0.719 |
| Invasive ductal carcinoma | 62 (92.5%) | 28 (93.3%) | 76 (89.4%) |  |
| Others | 5 (7.5%) | 2 (6.7%) | 9 (10.6%) |  |
| Invasive lobular carcinoma | 1 (1.5%) | 0 (0.0%) | 2 (2.4%) |  |
| Invasive papillary carcinoma | 2 (3.0%) | 1 (3.3%) | 0 (0.0%) |  |
| Microinvasive carcinoma | 2 (3.0%) | 0 (0.0%) | 5 (5.9%) |  |
| Metaplastic carcinoma | 0 (0.0%) | 0 (0.0%) | 1 (1.2%) |  |
| Invasive carcinoma with medullary features | 0 (0.0%) | 1 (3.3%) | 0 (0.0%) |  |
| Invasive carcinoma with neuroendocrine differentiation | 0 (0.0%) | 0 (0.0%) | 1 (1.2%) |  |
| Estrogen receptor |  |  |  | 0.001 |
| Expressed | 63 (94.0%) | 23 (76.7%) | 60 (70.6%) |  |
| Not expressed | 4 (6.0%) | 7 (23.3%) | 25 (29.4%) |  |
| Progesterone receptor |  |  |  | 0.002 |
| Expressed | 58 (86.6%) | 20 (66.7%) | 52 (61.2%) |  |
| Not expressed | 9 (13.4%) | 10 (33.3%) | 33 (38.8%) |  |
| Her-2 overexpression |  |  |  | 0.005 |
| Present | 6 (9.0%) | 10 (33.3%) | 26 (30.6%) |  |
| Absent | 61 (91.0%) | 19 (63.3%) | 58 (68.2%) |  |
| Equivocal | 0 (0.0%) | 1 (3.3%) | 1 (1.2%) |  |
| Subtype |  |  |  | <0.001 |
| Luminal A | 40 (59.7%) | 4 (13.3%) | 28 (32.9%) |  |
| Luminal B | 24 (35.8%) | 19 (63.3%) | 32 (37.6%) |  |
| Her-2 enriched | 1 (1.5%) | 4 (13.3%) | 13 (15.3%) |  |
| Triple-negative | 2 (3.0%) | 3 (10.0%) | 12 (14.1%) |  |
| Histologic grade |  |  |  | <0.001 |
| Grade 1 | 25 (36.3%) | 2 (6.7%) | 21 (24.7%) |  |
| Grade 2 | 33 (49.3%) | 5 (16.7%) | 31 (36.5%) |  |
| Grade 3 | 8 (11.9%) | 23 (76.7%) | 29 (34.1%) |  |
| Not reported | 1 (1.5%) | 0 (0.0%) | 4 (4.7%) |  |
| Lymphatic invasion |  |  |  | 0.019 |
| Present | 11 (16.4%) | 13 (43.3%) | 22 (25.9%) |  |
| Absent | 56 (83.6%) | 17 (56.7%) | 63 (74.1%) |  |
| Vascular invasion |  |  |  | 0.322 |
| Present | 5 (7.5%) | 0 (0.0%) | 6 (7.1%) |  |
| Absent | 62 (92.5%) | 30 (100.0%) | 79 (92.9%) |  |

**Supplementary Table 3.** Association between immunophenotype and clinical parameters.

| RFG | Wavelet | Feature | Coefficient |
| --- | --- | --- | --- |
| RFG_1_ | Db2 (ratio 1/2) | GLCM_correlation | -0.56754822 |
|  | Db2 (ratio 2/3) | NGTDM_complexity | -0.07508652 |
|  | Coif1 (ratio 2) | GLCM_sum_entropy | 0.54810966 |
|  | Coif1 (ratio 2) | NGTDM_complexity | -0.46805729 |
|  | Sym4 (ratio 2/3) | GLSZM_LZLGE | 0.10847101 |
|  | Sym4 (ratio 2/3) | GLSZM_LZHGE | -0.03217084 |
|  | Sym4 (ratio 3/2) | Global_variance | -0.07269342 |
| RFG_2_ | Db2 (ratio 1/2) | GLSZM_LZHGE | -0.19151188 |
|  | Coif1 (ratio 1/2) | GLCM_maxprob | -0.16563705 |
|  | Coif1 (ratio 2) | GLSZM_LZLGE | 0.35645318 |
|  | Sym4 (ratio 1/2) | GLCM_difference_entropy | 0.03883755 |
|  | Sym4 (ratio 3/2) | GLCM_IMC2 | -0.02537452 |
| RFG_3_ | None | GLCM_difference_entropy | 0.01796420 |
|  | None | GLCM_maxprob | 0.03596905 |
|  | None | GLSZM_LGZE | -0.25461542 |
|  | Db2 (ratio 2) | GLCM_maxprob | 0.10218814 |
|  | Sym4 (ratio 2/3) | GLCM_difference_entropy | 0.12967341 |
| RFG_4_ | Db2 (ratio 2/3) | Global_variance | 0.06256532 |
|  | Db2 (ratio 2/3) | GLCM_difference_entropy | 0.01333215 |
|  | Db2 (ratio 2) | GLCM_difference_entropy | 0.07290913 |
|  | Coif1 (ratio 1/2) | Global_variance | 0.05387090 |

**Supplementary Table 4.** Radiomic features selected by LASSO regression and their respective coefficient for predicting inflamed versus non-inflamed phenotype from whole tumor.

| Model | Model score or feature | Coefficient |
| --- | --- | --- |
| Scores | RM-whole_1_ | 0.6424617 |
|  | RM-whole_3_ | 0.2492717 |
|  | RM-whole_4_ | 0.4901712 |
| Features | RM-whole_1_, Db2 (ratio 1/2), GLCM_correlation | -0.058564466 |
|  | RM-whole_1_, Sym4 (ratio 3/2), Global_variance | -0.002479776 |
|  | RM-whole_3_, None, GLSZM_LGZE | -0.589146700 |
|  | RM-whole_3_, Db2 (ratio 2), GLCM_maxprob | 0.076042824 |
|  | RM-whole_4_, Db2 (ratio 2/3), GLCM_difference entropy | 0.177540941 |
|  | RM-whole_4_, Db2 (ratio 2), GLCM_difference entropy | 0.003808875 |

**Supplementary Table 5.** Model scores or features selected by LASSO regression and their respective coefficient for the combined models predicting inflamed versus non-inflamed phenotype from whole tumor.

| RFG | Wavelet | Feature | Coefficient |
| --- | --- | --- | --- |
| RFG_1_ | None | GLCM_IMC2 | 0.36076582 |
|  | None | NGTDM_strength | -0.35863589 |
|  | Db2 (ratio 1/2) | GLCM_energy | -3.99579390 |
|  | Db2 (ratio 1/2) | GLSZM_ZSV | -1.25137882 |
|  | Db2 (ratio 1/2) | NGTDM_coarseness | -2.94187759 |
|  | Db2 (ratio 1/2) | NGTDM_busyness | 1.35977314 |
|  | Db2 (ratio 2/3) | GLRLM_RLV | -1.01858984 |
|  | Db2 (ratio 3/2) | GLCM_entropy | -1.72317875 |
|  | Db2 (ratio 3/2) | NGTDM_coarseness | 6.45939590 |
|  | Coif1 (ratio 3/2) | GLSZM_ZSV | 0.08633585 |
|  | Coif1 (ratio 3/2) | NGTDM_coarseness | 0.77031059 |
|  | Coif1 (ratio 2) | Global_energy | -2.23659267 |
|  | Sym4 (ratio 1/2) | NGTDM_busyness | 3.07647699 |
| RFG_2_ | None | GLCM_IMC1 | 0.2476341 |
|  | None | NGTDM_busyness | 0.1639470 |
|  | Coif1 (ratio 1/2) | Global_energy | -0.1084947 |
|  | Sym4 (ratio 2) | GLSZM_ZSV | -0.9770190 |
| RFG_3_ | Db2 (ratio 1/2) | Global_uniformity | 0.6333171 |
|  | Db2 (ratio 2/3) | GLCM_agreement | 0.5242585 |
| RFG_4_ | None | Volume | 1.0556760 |
|  | Db2 (ratio 1/2) | NGTDM_coarseness | -0.2536303 |
|  | Coif1 (ratio 2) | Global_energy | -0.9789660 |
|  | Sym4 (ratio 2/3) | GLSZM_ZSV | -0.4272685 |
|  | Sym4 (ratio 2) | GLCM_IMC1 | 0.1629610 |

**Supplementary Table 6.** Radiomic features selected by LASSO regression and their respective coefficient for predicting immune-desert versus excluded phenotype from peripheral tumor.

| Model | Model score or feature | Coefficient |
| --- | --- | --- |
| RM-peri_SC_ | RM-peri_1_ | 0.5532408 |
|  | RM-peri_3_ | 0.1228667 |
| RM-peri_FC_ | RM-peri_1_, None, GLCM_IMC2 | -0.01088758 |
|  | RM-peri_1_, Db2 (ratio 1/2), NGTDM_busyness | 0.48700928 |
|  | RM-peri_1_, Coif1 (ratio 2), Global_energy | -0.36779554 |
|  | RM-peri_2_, None, GLCM_IMC1 | 0.20189324 |
|  | RM-peri_3_, Db2 (ratio 1/2), Global_uniformity | 0.46084793 |
|  | RM-peri_3_, Coif1 (ratio 3/2), GLCM_agreement | 0.45622116 |

**Supplementary Table 7.** Model scores or features selected by LASSO regression and their respective coefficient for the combined models predicting immune-desert versus excluded phenotype from peripheral tumor.

| Classification | Precision | Recall | F1-score |
| --- | --- | --- | --- |
| Immune-desert | 1.000 | 0.875 | 0.933 |
| Immune-excluded | 0.889 | 1.000 | 0.941 |
| Inflamed | 0.955 | 1.000 | 0.977 |
| Average | 0.948 | 0.958 | 0.950 |

**Supplementary Table 8.** Summary of immunophenotype prediction metrics based on scores from radiomic models.
